# Predictors associated with giving birth at home among women in Ethiopia

**DOI:** 10.11604/pamj.2025.50.1.38386

**Published:** 2025-01-02

**Authors:** Batholomew Chibuike James, Yenew Alemu, Matthew Esther Amairo, Kanokwan Chullapant, Emem Dickson Uzuagu, Chinazaekpere Mary Aroh

**Affiliations:** 1 Public Health Program, Sheffield Hallam University, Sheffield, United Kingdom,; 2 Department of Statistics, Injibara University, Injibara, Ethiopia,; 3 Department of Public Health Psychology, Highstone Global University, Texas, USA,; 4 Endocrinology Consultation Phatthalung Hospital, Phatthalung Province, Thailand,; 5 Ministry of Public Health, Doha, Qatar,; 6 Staff Nurse, Royal Berkshire NHS Foundation Trust, London Rd, Reading RG1 5AN, United Kingdom

**Keywords:** Predictor, association, giving birth at home, binary logistic regression, Ethiopia

## Abstract

**Introduction:**

maternal mortality is a worldwide community health concern. Home deliveries are common in Ethiopia, and most births occur at home without the assistance of health experts. Maternal mortality reduced in recent decades but still has a very high maternal mortality rate in Ethiopia. Therefore, this study aimed to identify the risk factors surrounding giving birth at home among women in Ethiopia.

**Methods:**

this study's data source was the 2019 Ethiopian Demographic and Health Survey (EDHS). A total of 23,007 women who met the study's objective and criteria were included. A binary logistic regression model and a multistage stratified sampling technique were used.

**Results:**

women living in a rural area (AOR = 2.135, 95% CI: 1.805, 2.525), women in the middle (AOR = 0.670, 95% CI: 0.590, 0.760), and wealth index (AOR = 0.375, 95% CI: 0.326, 0.430), mothers who attended primary education (AOR = 0.819, 95% CI: 0.733, 0.915), secondary and above (AOR = 0.388, 95% CI: 0.303, 0.496), 4-6 living children (AOR= 0.780, 95% CI: 0.160, 0.873), mother age from 21-30 (AOR= 0.291, 95% CI: 0.243, 0.349) and mother age from 31 and above (AOR = 0.074, 95% CI: 0.060, 0.091) were significantly associated predictors for giving birth at home in Ethiopia.

**Conclusion:**

we discovered that geographical region, place of residence, education level, marital status, age of mother at first birth, mother age group, number of living children, religion, and wealth index were significantly associated predictors of giving birth at home among Ethiopian women.

## Introduction

Pregnancy occurs when one or more children develop inside a woman [1]. Women give birth commonly at home or in health institutions. Home deliveries raise the possibility of women's mortality [2]. Maternal mortality is a worldwide community health concern; around 810 maternal deaths happened daily in 2017 [3]. About 830 women are dying per day due to childbirth. The estimated average maternal mortality rates were 216 and 546 per 100,000 births, globally and sub-Africa, respectively. Ethiopia has one of the highest maternal mortality rates in the world [4]. In the world, every year, approximately 303,000 women die due to problems with childbearing. Around 99% of the total parental deaths occurred in low-income countries. Annually, 13,000 maternal deaths occur in Ethiopia [5].

One-third of births in the world take place at home. More than 50% of deliveries in Africa occur at home without skilled health workers. Ethiopian maternal death was 412 per 100,000 in 2016 [6]. Home deliveries are common in Ethiopia; around 94% of births occur at home without skilled health professionals' assistance [5]. Maternal mortality has reduced in recent decades, but it still has a very high maternal mortality rate (412 deaths per 100,000 live births) [7]. Factors that prevent women from receiving adequate health care during pregnancy and childbirth include lack of access to transportation and child care, unmet needs for work leave to attend appointments, lack of knowledge of pregnancy, and inability to find a clinic or obtain a timely selection [4].

Muslim and Protestant religions were positively related to home delivery. At the same time, parents who had a medium and above wealth index of households and women who attended primary and above education were negatively associated with home delivery among women in Ethiopia [8]. In addition, home delivery mainly occurred to rural parents, uneducated women, aged women, and mothers with low-income families [9]. Numerous studies have been conducted about giving birth at home. Rural residence and no maternal education are associated with maternity places of delivery at home [10]. Lower maternal education rates positively correlate with home birth opportunities [11]. In addition, the maternal education level influences the maternal giving birth at home [12,13]. Another factor that influences home delivery is that religion significantly affects mothers' birthplace [13,14].

Most maternal deaths are caused by obstetric care. If giving birth at home is not done by professionals, it increases the risk of infection, postpartum haemorrhage, and the spread of HIV/AIDS. The burden of home childbirth, primarily unsupervised, is not only limited to maternal health problems but also ends in maternal and infant illness and death [15]. Ethiopian health institutional deliveries occurred around 48% in 2019 due to a lack of transportation and the scarcity of appropriate health facilities. Home delivery is still public, especially in hard-to-reach areas [16]. This indicates that maternal home delivery and maternal death are still high in Ethiopia. To decrease maternal and infant mortality, this study explores to identify the risk factors of giving birth at home among women in Ethiopia.

## Methods

**Source of data:** the Ethiopian Demographic and Health Survey conducted in 2019 by the Central Statistical Agency was the source of this study data. The Data collection was held from March to June 2019. With the availability of the data on the Ethiopia DHS website [17] and permission fully granted, we analysed the incidence of 23,007 women between the ages of 15 and 49 years.

**Study design:** the 2019 Ethiopian Demographic and Health Survey sample was selected in two stages. Each region was divided into urban and rural areas and provided 21 samples. Samples of enumeration areas at each level were chosen individually in two steps. According to the administrative divisions at different levels, there was an apparent breeding and proportional distribution at each lower organisational level before identifying the sample framework in each sample and using the opportunity at the initial level proportional to size selection. In the first step, 305 enumeration areas were randomly selected in each sample size corresponding to the enumeration area size. In the second round of voting, a fixed number of 30 households in a cluster were selected equally from the newly formed family list. All women aged 15-49 for selected families, permanent residents, or overnight visitors before the study are eligible for an interview [16].

**Sample size determination:** this study is based on secondary data from the 2019 Ethiopia Demographic and Health Survey (EDHS, 2019) conducted by the Central Statistics Agency in collaboration with the Ministry of Health and other donor agencies. This study included a sample size of 23,007 childbearing women aged 15 to 49. The data collected during the current study are publicly available on the Ethiopian Demographic and Health Survey website [17].

**Dependent variable:** the response to this study was a place of the delivery of mothers that if a mother gave birth at home, recorded by 1 and 0 if at the health institution.

**Independent variable:** the explanatory variables included are types of the place of residence (urban, rural), Region (Tigray, Afar, Amhara, Oromia, Somali, Benishangul, SNNPR, Gambela, Harari, Addis Ababa, Dire Dawa), Religion (Orthodox, Muslim, others), types of birth (single, multiple), marital status (married, unmarried), wealth index (poor, medium, rich), mothers education level (no educated, primary, secondary and above), age of mothers at the first birth (18 and below,19 and above), mother age group (20 and below, 21-30, 31 and above) and number of living children (3 and below, 4-6, 7 and above).

**Ethics approval and consent to participate:** ethical approval and willingness to participate in this study included using a designated database previously published by the Central Statistics Agency, so ethical approval and participatory agreement were not required.

**Data management and analysis methods:** Statistical Package for Social Sciences (SPSS) software version 26 was used for data coding and analysis. Based on the available data, descriptive analyses were performed using both frequency and percentage variables and independent variables. Logistics has been used to identify the causes of home birth risk among women in Ethiopia. The Homer-Lemeshow test is an excellent statistical test to suit logistical regression models [18]. All hypotheses for determining differences, associations, and relationships were significantly reduced by p-value <0.05.

## Results

**Descriptive characteristics of the study respondents:** a total of 23,007 women were included in this study. 2,881 (12.5%) gave birth at home, while 20,126 (87.5%) delivered their babies to health facilities (Table 1). Of women from rural settlements, 2,657 (11.5%) had their babies at home, while 67.1% delivered them at health institutions. Only 224 (1%) of those living in urban areas have their babies at home, while 20.4% deliver at health facilities (Table 2). Of the entire women, only 9.1% and 7.4% of uneducated women gave birth at home, and those that gave birth to maternal age at first 18 and below, respectively. In addition, the rich index of this study shows that 9% of poor women give birth at home, while only 1.8% and 1.7% of women in middle and upper-income levels give birth at home (Table 2) 0.1% of Addis Ababa City Administration and 0.6% of Dire Dawa City Administration women deliver at home. On the other hand, 0.6% from Tigray, 2.2% from Afar, 1.1% from Amhara, 1.8% from Oromia, 2.2% from Somali, 0.8% from Benishangul-Gumuz, 1.6% from SNNPR, 0.9% from Gambella and Harari from 0.7% of women deliver at home (Table 2). In the bi-variable logistic regression model, Religion, place of residence, marital status, region, wealth index, maternal education level, maternal age group, maternal age at birth, and the number of children were significant effects on the place of delivery at p-value <0.25 and fitted the multi-variable logistic regression analysis. Based on the data, Hosmer and Lemeshow´s result, p-value =0.1504> 0.05 at a 5% significance level, indicates that the binary logistic regression model is adequate.

**Table 1:** frequency and percentage of place of delivery among women

		Frequency	Percentage (%)
Place of delivery	Health institution	20,126	87.5
	home	2,881	12.5
	Total	23,007	100.0

**Table 2:** frequency, percentage, and cross tabulation of independent variables with the place of delivery

		Place of delivery				Chi-square statistic	p-value
		Home		Health institution			
		frequency	%	frequency	%		
**Region**	Tigray	144	0.6	1,667	7.2	770.023	0.000
	Afar	500	2.2	1,448	6.3		
	Amhara	247	1.1	2,224	9.7		
	Oromia	421	1.8	2,780	12.1		
	Somali	517	2.2	1,688	7.3		
	Benshangul-gumuz	180	0.8	1983	8.6		
	SNNPR	362	1.6	2,781	12.1		
	Gambela	204	0.9	1,771	7.7		
	Harari	155	0.7	1,487	6.5		
	Addis Ababa	14	0.1	792	3.4		
	Dire Dawa	137	0.6	1,505	6.5		
**Types of place of residence**	Urban	224	1	4,698	20.4	363.211	0.000
	rural	2,657	11.5	15,428	67.1		
**Religion**	Orthodox	599	2.6	6,400	27.8	200.733	0.000
	Muslim	1,671	7.3	9,011	39.2		
	others	611	2.7	4,715	20.5		
**Types of birth**	Single	2,816	12.2	19,630	85.3	0.460	0.498
	multiple	65	0.3	496	2.2		
**Marital status**	married	2,708	11.8	17,647	76.7	98.475	0.000
	unmarried	173	0.8	2,479	10.8		
**Wealth index**	poor	2,080	9	9,150	39.8	797.650	0.000
	medium	408	1.8	3,423	14.9		
	rich	393	1.7	7,553	32.8		
**Mother4s education level**	No education	2083	9.1	13,500	58.7	106.429	0.000
	primary	707	3.1	4,879	21.2		
	Secondary and above	91	0.4	1,747	7.6		
**Number of living children**	3 and below	1,208	5.3	5,511	24	298.297	0.000
	4-6	1,226	5.3	9,470	41.2		
	7 and above	447	1.9	5,145	22.4		
**Age of the mother at the first age**	18 and below	1697	7.4	13,176	57.3	47.517	0.000
	19 and above	1,184	5.1	6,950	30.2		
**Mothers age group**	20 and below	319	1.4	416	1.8	1656.395	0.000
	21-30	1640	7.1	6,155	26.5		
	31 and above	922	4	13,555	58.9		

**Factors related to giving birth at home among women in Ethiopia:** the geographical region was important for home delivery among women in Ethiopia. The odds of delivery at home for mothers living in Afar, Amhara, Oromia, Somali, SNNPR, Harari and Dire Dawa were 2.9 (p-value= 0.000), 1.3 (p-value= 0.034), 1.8 (p-value= 0.000), 3.0 (p-value= 0.000),1.40 (p-value= 0.009), 1.9 ((p-value= 0.000),)and 1.4 (p-value= 0.042) times more than mothers living in Tigray region, respectively (Table 3). Also, the odds of home delivery for mothers between the ages of 21 and 30 were 0.3 (p-value = 0.000) times less than for mothers aged 20 and under. For mothers 31 and older, the risk of having a home birth was 0.1 (p-value= 0.000) times more likely for mothers aged 20 and under (Table 3). In this study, we observed that place of residence significantly affected home delivery among women in Ethiopia, meaning the odds of home delivery for mothers living in rural areas was 2.1 (p-value= 0.000) times more than for mothers living in urban areas. In addition, home birth opportunities for mothers who follow Muslims were 0.8 (p-value= 0.017) times less than for mothers who followed orthodoxly (Table 3).

**Table 3:** predictors of home delivery among women in Ethiopia

Place of delivery	AOR	Std.Err	z	P>|z|	95% C.I.	
**Region, Tigray(ref.)**						
Afar	2.938	0.406	7.79	0.000*	2.240	3.852
Amhara	1.281	0.149	2.13	0.034*	1.019	1.610
Oromia	1.806	0.229	4.67	0.000*	1.409	2.314
Somali	2.988	0.411	7.95	0.000*	2.2812	3.914
Bsnshangul-gumuz	0.875	0.118	-0.99	0.322	.672	1.139
SNNPR	1.399	0.180	2.61	0.009*	1.087	1.80
Gambela	1.304	0.182	1.90	0.057	0.992	1.715
Harari	1.886	0.290	4.12	0.000*	1.395	2.550
Addis Ababa	0.665	0.200	-1.36	0.175	.368	1.199
Dire Dawa	1.373	0.214	2.03	0.042*	1.011	1.865
**Types of places of residence, urban (ref.)**						
Rural	2.135	0.183	8.85	0.000*	1.805	2.525
**Religion, orthodox (ref.)**						
Muslim	0.809	0.072	-2.40	0.017*	0.680	0.962
Others	1.089	0.097	0.96	0.335	0.915	1.296
**Marital status, married (ref.)**						
Unmarried	0.587	0.052	-6.01	0.000*	0.494	0.699
**Wealth index, poor (ref.)**						
Medium	0.670	0.043	-6.19	0.000*	0.590	0.760
Rich	0.375	0.027	-13.87	0.000*	0.326	0.430
**Mother's education level, no education (ref.)**						
Primary	0.8189	0.047	-3.52	0.000*	0.733	0.915
Secondary and above	0.388	0.049	-7.55	0.000*	0.303	0.496
**Number of living children, 3 and below (ref.)**						
4-6	0.780	0.045	-4.35	0.000*	0.698	0.873
7 and above	0.738	0.059	-3.82	0.000*	0.631	0.862
**Age of mothers at 1st birth, 18 and below (ref.)**						
19 and above	1.871	0.091	12.87	0.000*	1.701	2.059
**Mother’s age group, 20 and below (ref.)**						
21-30	0.291	0.027	-13.30	0.000*	0.243	0.349
31 and above	0.074	0.008	-24.31	0.000*	0.060	0.091
Constant	0.469	0.074	-4.83	0.000	0.345	0.638

AOR: adjusted odd ratio; C.I: confidence interval; SNNPR: Southern Nations, Nationalities, and Peoples' Region

Furthermore, the results of this study show that the wealth index significantly impacted home delivery among women in Ethiopia. Women with medium and rich wealth indexes were 0.7 (p-value= 0.000) and 0.4 (p-value= 0.000) and less likely to deliver at home from poor economic conditions, respectively. Maternal education hurts home delivery among women in Ethiopia. The odds of home birth among women in primary and secondary and above education were 0.8 (p-value= 0.000) and 0.4 (p-value= 0.000) times less than uneducated women, respectively (Table 3). According to some living children, the odds of home delivery for mothers with 4-6 children were 0.8 (p-value= 0.000) times more than for mothers with three and below children. In addition, the odds of home delivery for mothers with seven and above children were 0.7 (p-value= 0.000) times less than for mothers with three and below children. Moreover, mothers´ age at first birth is a significant factor for home delivery among women in Ethiopia. The odds of home delivery for mothers aged at first birth 19 and above was 1.9 (p-value= 0.000) times more than the mother´s age at first birth 18 and below. It is also important to know that marital status was a significant predictor of home delivery in Ethiopia. The probability of having a home birth for unmarried mothers was 0.6 (p-value= 0.000) times less than for married mothers (Table 3).

## Discussion

This study aimed to identify the risk factor of the place of delivery of women in Ethiopia using a logistic regression model. Wealth index, region, religion, marital status, mother's age, maternal level of education, place of residence, mother's age at the first birth, and number of living children were strong predictors of home delivery. The findings show that women in the middle and high-income bracket were less likely to give birth at home compared to women from lower-income groups. This could be associated to lesser transportation problems faced by women in higher income brackets, facilitating their access to health services more often. In addition, their financial stability enables this group of women to seek institutional health care more consistently. This finding aligns with studies conducted in Ethiopia and Eritrea. [8,9,19]. The finding of this study shows that place of residence was a significant predictor for home delivery among women in Ethiopia. The odds of home delivery for mothers living in rural areas were higher than for mothers living in urban areas. This result is expected. The reason may be that rural areas do not have enough infrastructure and health services, so pregnant women may not get transport facilities (ambulances). This result is consistent with other findings [9,10,15,20-23]. This study also revealed that the mother´s education level was another predictor of home delivery. The conclusion was that mothers with no formal education were more likely to deliver at home than those educated mothers (primary and above education level of mothers). The reason may be that educated women have enough knowledge about childbirth, visiting health facilities, and accepting health professionals' advice. This is in line with other studies [8-11,13,15,24,25]

This study also shows that women between the ages of 21 and above are less likely to have children at home. Women aged 21 and over were less likely to give birth at home than women aged 20 and under. Possible explanations are that older women are less likely to have planned pregnancies and may have more knowledge of cultural values and risk factors. In addition, they have more childbirth experience. The finding does not coincide with previous studies [26,27]. But this result is consistent with other studies [15]. The number of living children was statistically significantly associated with giving birth at home. The number of living children was very strongly associated with home delivery. The odds of giving birth at home were lower for women with 4 -6 living children than for women with three and below living children. This finding is inconsistent with studies conducted in Ghana [27].

The finding of this study further revealed that marital status was a significant factor in giving birth at home. Unmarried women were less likely to have children at home than married women. This finding is not in line with studies in Zambia [28]. In addition, the odds of home delivery for mothers aged 19 and above were more than for mothers aged 18 and below. This result is unexpected. The reason may be that mothers aged at first birth 19 and above are more likely to have unplanned pregnancies and less likely to have signs of pregnancy. Afar, Amhara, Oromia and SNNPR were areas at high risk for giving birth at home. This result is consistent with other studies [8]. In addition, Harari and Dire Dawa were at increased risk of giving birth at home in Ethiopia. This study is contrary to other findings [8]. Somalis were the most vulnerable regions compared to Tigray. In addition, home birth opportunities for mothers who are Muslim were less than for mothers who practice the Orthodox faith. The main reason for this may be that conventional gives further education about giving birth at home in addition to theological parts. This study´s finding was contrary to other related studies that showed that religion influenced a mother´s choice of delivery [8].

**Strength and limitation:** the strength of this study is that the selected women were the study population, and the selection process was well-designed. The study only interviewed surviving women, except barren women, women with mental illness, and hearing loss women. Therefore, no data were available for the children of women who had died. The data used in this study are from the EDHS 2019. Thus, the results may not necessarily reflect the current situation in Ethiopia.

## Conclusion

This study shows that 12.5% of mothers give birth at home. The region, place of residence, education level, marital status, mother´s age at first birth, mother´s age group, number of living children, religion, and wealth index were significant predictors of giving birth at home among women in Ethiopia. The government and public health institutions should provide a variety of care to reduce giving birth at home among rural women, uneducated women, young age group of women, poor women and women living in Afar, Amhara, Oromia, SNNPR, Dire Dawa, Harari and Somali. Strategies should be developed to expand access to institutional delivery services among at-risk groups.

### What is known about this topic

- Maternal mortality is a worldwide community health concern;
- Home deliveries are common in Ethiopia, and most births occur at home without the assistance of health experts;
- The burden of home childbirth is not only limited to maternal health problems but also ends in maternal and infant illness and death.

### What this study adds

- The majority of women living in rural areas delivered their babies at home; educated women with fewer children and medium to higher income were less likely to deliver at home.
- Unmarried women were less likely to have children at home than married women;
- Afar, Amhara, Oromia, Harari, Dire Dawa and SNNPR regions were high for giving birth at home compared to the reference category.
